# Nanoparticle-Based and Bioengineered Probes and Sensors to Detect Physiological and Pathological Biomarkers in Neural Cells

**DOI:** 10.3389/fnins.2015.00480

**Published:** 2015-12-18

**Authors:** Dusica Maysinger, Jeff Ji, Eliza Hutter, Elis Cooper

**Affiliations:** ^1^Department of Pharmacology and Therapeutics, McGill UniversityMontreal, QC, Canada; ^2^Department of Physiology, McGill UniversityMontreal, QC, Canada

**Keywords:** gold nanoparticles, nanosensors, microglia, neurons, quantum dots, Ca^2+^, MMP, caspases

## Abstract

Nanotechnology, a rapidly evolving field, provides simple and practical tools to investigate the nervous system in health and disease. Among these tools are nanoparticle-based probes and sensors that detect biochemical and physiological properties of neurons and glia, and generate signals proportionate to physical, chemical, and/or electrical changes in these cells. In this context, quantum dots (QDs), carbon-based structures (C-dots, grapheme, and nanodiamonds) and gold nanoparticles are the most commonly used nanostructures. They can detect and measure enzymatic activities of proteases (metalloproteinases, caspases), ions, metabolites, and other biomolecules under physiological or pathological conditions in neural cells. Here, we provide some examples of nanoparticle-based and genetically engineered probes and sensors that are used to reveal changes in protease activities and calcium ion concentrations. Although significant progress in developing these tools has been made for probing neural cells, several challenges remain. We review many common hurdles in sensor development, while highlighting certain advances. In the end, we propose some future directions and ideas for developing practical tools for neural cell investigations, based on the maxim “Measure what is measurable, and make measurable what is not so” (Galileo Galilei).

## Introduction to sensors and their applications in neuroscience

**Figure 1:**
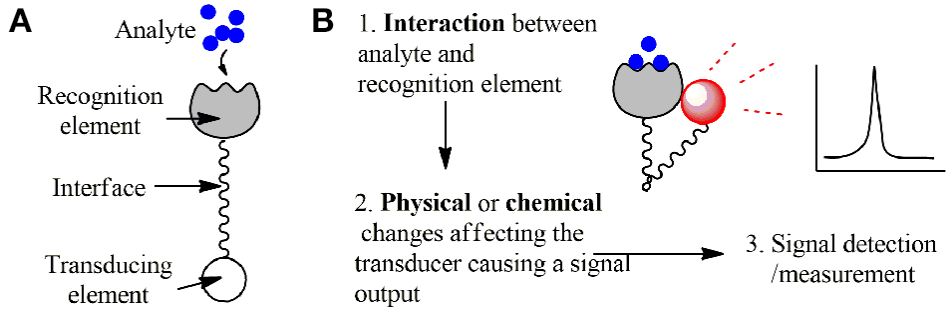
**A simplified presentation of a sensor's components. (A)** Components of a sensor. **(B)** Steps of the biosensing process.

Recent advances in nanotechnology have provided neuroscientists with powerful new tools. Among these are some probes and nanosensors constructed with materials ranging from organic molecules to metallic nanostructures to engineered fluorescent proteins. Probes are defined as small devices used to explore, investigate or measure something by penetrating or being placed in the cells, cell lysate, and extracellular media. A sensor is an assembly required to detect and communicate a particular event: a device or biological structure which (i) recognizes an entity of interest (e.g., molecules, ions, or physical changes such as temperature) and (ii) transduces an event of recognition into a measurable signal. Recognition and signal transduction is followed by signal detection in the process of biosensing (see Figure 1). In case of nanosensors, the terms “probe” and “sensor” often overlap, because nanostructures are penetrating or “in-place” devices and usually serve as a recognition element, a transducer and even a signal amplifier at the same time.

Neural cells respond to dangers and noxious stimuli with a cascade of events involving diverse classes of molecules and ions. So far, probes and sensors have been designed to detect proteins such as signaling molecules and enzymes, ions (e.g., Ca^2+^, K^+^, Na^+^, H^+^, or pollutants such as Hg^2+^ and Cd^2+^), simple molecules which are critically important for cell metabolism (e.g., glucose, lipids), DNA, changes in pH, redox, and neurotransmitters as well as morphological (e.g., shape and size of neuronal and glial soma, neurites and post-synaptic spines) and functional changes (e.g., action potentials, mitochondrial potential, inter and intracellular organellar communication). “Biofriendly” nanosensors seem to be suitable candidates for intracellular sensing since they are significantly smaller than the size of cells, and chemically inert so as not to interfere with cellular functions during measurements (Howes et al., 2014). However, only few nanosensors have been tested in neural cells. Recent review by Howes et al. (2014) provides a general overview of nanoparticle-based sensors, their use and limitations in biology.

In this review, we focus on several nanoparticle-based and bioengineered sensors mainly for proteases (e.g., metalloproteases and caspases) and biomolecules implicated in disrupted homeostasis in neural cells. To highlight the advantageous features of these nanoparticle-based tools, as well as to discuss critically some of their limitations, we have chosen a few examples from research on inflammatory processes in the nervous system (e.g., caspase-1). First, we provide a brief overview of nanostructures used as probes or components of nanosensors, then we discuss nanosensors and genetically engineered sensors for proteases and aromatase. We then highlight probes and sensors for ions and ion channels focusing on calcium, a principal regulator of many neuronal functions. We provide examples of neural stimulation using nanostructures. To emphasize the complexity of the sensing in the nervous system, we comment on glia as “natural biological sensors” and finally summarize current approaches and challenges in designing suitable nanostructured sensors to detect biomarkers under physiological and pathological conditions.

## Nanoparticle-based and bioengineered sensors for neural cells

Many neurological impairments are associated with different chemical and physical insults that disrupt cell homeostasis. Numerous attempts has been made to follow the progression of pathological processes non-invasively, but only a few established and commonly used bioengineered sensors are able to monitor biochemical and morphological changes in neural cells longitudinally. Moreover, no nanostructured materials are dedicated solely to the development of nanosensors for the detection and monitoring of changes specifically in neural cells, because: (1) noxious stimuli are deleterious to various cell types aside from neural cells, (2) cell responses to danger and harmful stimuli are often similar in different cell types, and (3) availability of primary human and animal neural cells is limited. Examples of organic and metal-based nanostructures for measurement of biomolecules in cells and tissues of the nervous system are provided in Table 1.

**Table 1:** **Examples of physiologically relevant molecules in neural cells or tissues measured by various nanoparticle-based probes and sensors**.

**Analyte**	**Nanoparticle type**	**Cell/tissue type**	**Detection principle**	**References**
Oxygen	QD	Hippocampal slice	The sensor is based on FRET between QDs and a fluorescent dye. The QDs are immobilized in a thin layer of polymer matrix, which is deposited either on glass (hippocampal slices are then placed directly on top of the film) or on a microelectrode (placed into the extracellular matrix of the CA1 stratum pyramidale). Luminescence intensity ratio between the QD and dye changes according to the O_2_ content in the artificial cerebral spinal fluid bathing the brain slices.	Ingram et al., 2013
Oxygen	Anionic NPs of acrylic co-polymer	Primary neural cells, multi-cellular aggregates (3D spheroids) and cultured organotypic brain slices	NPs are impregnated with a phosphorescent dye, internalized by endocytosis, and are transported to lysosomes. The phosphorescence lifetime of the dye correlates with the intracellular O_2_ concentration.	Dmitriev et al., 2015
Reactive oxygen species	AuNP	Post-ischemic rat brains	Fluorescein-labeled hyaluronic acids (HA) are immobilized on AuNPs. The probes are injected locally into the focal ischemic brain of a brain stroke animal model. When ROS degrades the HA, the fluorescence dye is released from the AuNPs and is unquenched.	Hyun et al., 2013
Sodium	PAMAM-CG	Primary neurons	A sodium dye is encapsulated in a PAMAM dendrimer nanocontainer. When loaded into neurons in live brain tissue, it homogenously fills the entire cell volume, including small processes. The fluorescence intensity correlates with sodium concentration.	Lamy et al., 2012
Nitric oxide	Carbon nanotubes	Microdialysate from rat brain (*in vivo*)	Hemin and multi-wall carbon nanotubes are covalently attached to chitosan; the chitosan is electrodeposited on the surface of carbon fiber microelectrodes. Exogenously applied NO is measured by square wave voltammetry in the rat brain *in vivo*.	Santos et al., 2013
Ascorbate	Carbon nanotubes	Microdialysate from rat brain (*in vivo*)	A glass carbon electrode modified with heat-treated single-walled carbon nanotubes (SWNTs) is capable of electro-oxidizing the ascorbic acid (AA). Brain microdialysate is directly delivered into a thin-layer radial electrochemical flow cell for the continuous measurement of AA concentration.	Liu et al., 2008
Glucose	AuNP	Microdialysate from rat brain	ssDNA modified AuNPs aggregate in the presence of glucose resulting in an absorbance peak shift (*in vitro* colorimetric detection).	Jiang et al., 2010
Cysteine	AuNP	Microdialysate from the striatum of rat brain	Cysteine causes the aggregation of citrate stabilized AuNPs, resulting in an absorbance peak shift (*in vitro* colorimetric detection).	Qian et al., 2012
Lead	Graphene quantum dot	Cerebrospinal fluid of rats	A rigid structure is formed between tryptophan and GQD-DMA conjugates in the presence of Pb^2+^ (acting as a cross-linker). The resulting increase in fluorescence allows for the detection of Pb^2+^ in brain microdialysate (*in vitro* measurement).	Qi et al., 2013
Inducible Nitric Oxide Synthase	AuNP	Lysed A172 neuronal cell	An electrode is modified with AuNPs and anti-iNOS antibodies. The attachment of iNOS causes changes in chronoamperometric measurements in a concentration dependent manner.	Koh et al., 2011
Caspase-1	QD	Glial cells	QDs and a fluorescent dye are linked through a caspase-1 substrate peptide (FRET condition). In the presence of caspase-1 activity, FRET is lost and fluorescence ratios change (*in vitro* measurements from cell lysates).	Moquin et al., 2013a
Tumor specific receptor, EGFR	QD	Glioma (cell-culture, animal model and *ex vivo* human tumor biopsies)	Living glioma and normal cells or tissue biopsies are incubated with QDs coupled to EGF and/or monoclonal antibodies against EGFR). Visualization is done by various microscopies.	Kantelhardt et al., 2010
Tumor specific receptor, EGFR	QD	Medulloblastoma and glioma cancer cells	EGF receptors are labeled with QD-antiEGFR conjugates. QDs are internalized together with the receptors, quantitatively revealing the population of activated EGFR.	Dudu et al., 2011
Metabotropic glutamate receptor 1a (mGluR1a)	AuNP	Cultured hippocampal pyramidal cells	AuNPs are used as cell surface labels to evaluate the somatodendritic and axonal distribution of mGluR1a.	Fraire et al., 2014
NMDA and AMPA surface receptors	QD	Hippocampal neuron cultures	Using QDs coupled to antibodies directed against the N-terminus of the NR1 subunit of NMDA receptors or the GluR2 subunit of AMPA receptors, single QDs are tracked in the extrasynaptic and synaptic membranes of hippocampal neurons.	Michaluk et al., 2009
Tumor microenvironment (MMP-2, low pH)	AuNP	Glioma, in spheroids (C6) and animals	Doxorubicin (DOX) and Cy5.5-decorated AuNP are integrated into matrix metalloproteinase-2 (MMP-2) degradable gelatin nanoparticles. DOX and Cy5.5 linked to AuNPs through a hydrazine bond to enable pH-triggered cargo release. Active glioma targeting is enabled using surface modification with RRGD, a tandem peptide. At glioma sites, MMP-2 degrades the gelatin nanoparticles and the release of DOX and Cy5.5 is triggered by low pH.	Ruan et al., 2015

*QD, Quantum dots; AuNP, Gold nanoparticles; PAMAM-CG, polyamidoamine dendrimer course grain*.

Most of the nanostructures shown in Table 1 have to be internalized by cells to detect and measure the intracellular changes of biomolecules. Endocytosis and cooperative transmembrane penetration of nanoparticles are the ways by which nanoparticles can enter cells (Cleal et al., 2013; Yameen et al., 2014; Shang et al., 2014a,b; Beddoes et al., 2015; Kafshgari et al., 2015; Tan et al., 2015; Zhang et al., 2015b). Although endocytosis of nanoparticles (NP) has been studied for quite a while, the precise mechanisms are still not defined due to the complexities of these processes and technical problems associated with them. The emerging picture is that different cells engage in different, and sometimes complementary routes of internalization. This conclusion was derived from studies employing pharmacological agents, knockdown approaches using siRNA and knockout technologies taking advantage of selective deletion of the selected protein anticipated to be involved in the endocytic process (e.g., Iversen et al., 2011). To enhance the chance of nanoparticle entry into cells, various surface modifications were made including the attachment of cell penetrating peptides (Jones and Sayers, 2012; Onoshima et al., 2015). Penetration of lipid bilayers in membranes (cooperative transmembrane penetration) is considered an alternative pathway of NP entry aside from endocytosis (Zhang et al., 2015b). Translocation efficiency via this non-endocytic route depends on NP quantity, NP surface properties, NP aggregation and agglomeration state as well as the properties of cell membranes. It is conceivable that cooperative penetration contributes to the internalization of NP-based sensors. Simulation studies by Zhang et al. suggest that the particle quantity, NP surface properties and membrane structures are closely linked with NP-membrane forces and efficiency of NP penetration (Zhang et al., 2015b).

Traditionally, detection and quantification of intracellular analytes were achieved by employing fluorescent dyes (probes; Haugland, 2005; Lakowicz, 2006) and examples are listed in Table 2.

**Table 2:** **Common molecular probes for the detection of intracellular biomolecules, ions and reactive oxygen species in neural cells**.

**Name of Probe**	**Analyte**	**Function**	**Ex/Em (nm)**
CM-H2DCFDA	ROS	Indicator of ROS status in cells. Oxidation of DCF by ROS increases fluorescence signal	492–495/517–527
DHE	ROS	Superoxide indicator. Oxidation of hydroethidine causes it to intercalate DNA and switch from blue to red fluorescence	518/605 (DNA bound)
Mito-SOX	ROS	Mitochondrial superoxide indicator. Localizes to mitochondria and reactive to superoxides. Oxidized Mito-SOX excites at ~400 nm. Not sensitive to other reactive oxygen or reactive nitrogen species	510/580
C-11 Bodipy 581/591	Lipid Peroxidation	Sensor for lipid peroxidation. Oxidation of C-11 Bodipy changes fluorescence emission spectra from red to green	Normal: 581/591 Oxidized: ~ 485/520
Fluo-3	Ca^2+^	Calcium indicator, increase fluorescence upon calcium binding	506/526
Calcium Green^TM^-1	Ca^2+^	Calcium indicator, increase fluorescence upon calcium binding. Brighter resting cell fluorescence than fluo-3 but less phototoxic than fluo-3	506/531
Fura Red	Ca^2+^	Binding to calcium decreases fluorescence emission when excited at 488 nm. Allows ratiometric measurements of calcium levels by measuring the emission when exciting at 420 nm and 488 nm in the same field	Free: 488/670 Bound: 420/670
SBFI/PBFI	Na^+^/K^+^	Sodium/potassium ion sensitive fluorescent probe. Comprised of a benzofuranyl fluorophore linked to a crown ether chelator. The crown ether pore size is ion selective. Fluorescence increases upon ion binding	340, 380/500
Sodium Green^TM^	Na^+^	Sodium ion sensitive fluorescent probe. Greater fluorescence quantum yield then SBFI. Fluorescence increases upon Na^+^ binding	510/530
MitoImage^TM^ NanO2 Probe	O_2_	Probe phosphorescence is reversibly quenched by O_2_ inside cells. Probe signal decreases with increasing O_2_ and increases with decreasing O_2_	385/640

These dyes are non-invasive probes and relatively simple-to-follow, but rapid bleaching, the requirement of organic solvents (dissolution of lipophilic dyes) or the unpredictable interactions with intracellular molecules often limit their usefulness. QDs, carbon nanomaterials and PEBBLEs are good alternatives to avoid some of the problems associated with fluorescent dyes.

### QDs

**Figure 2:**
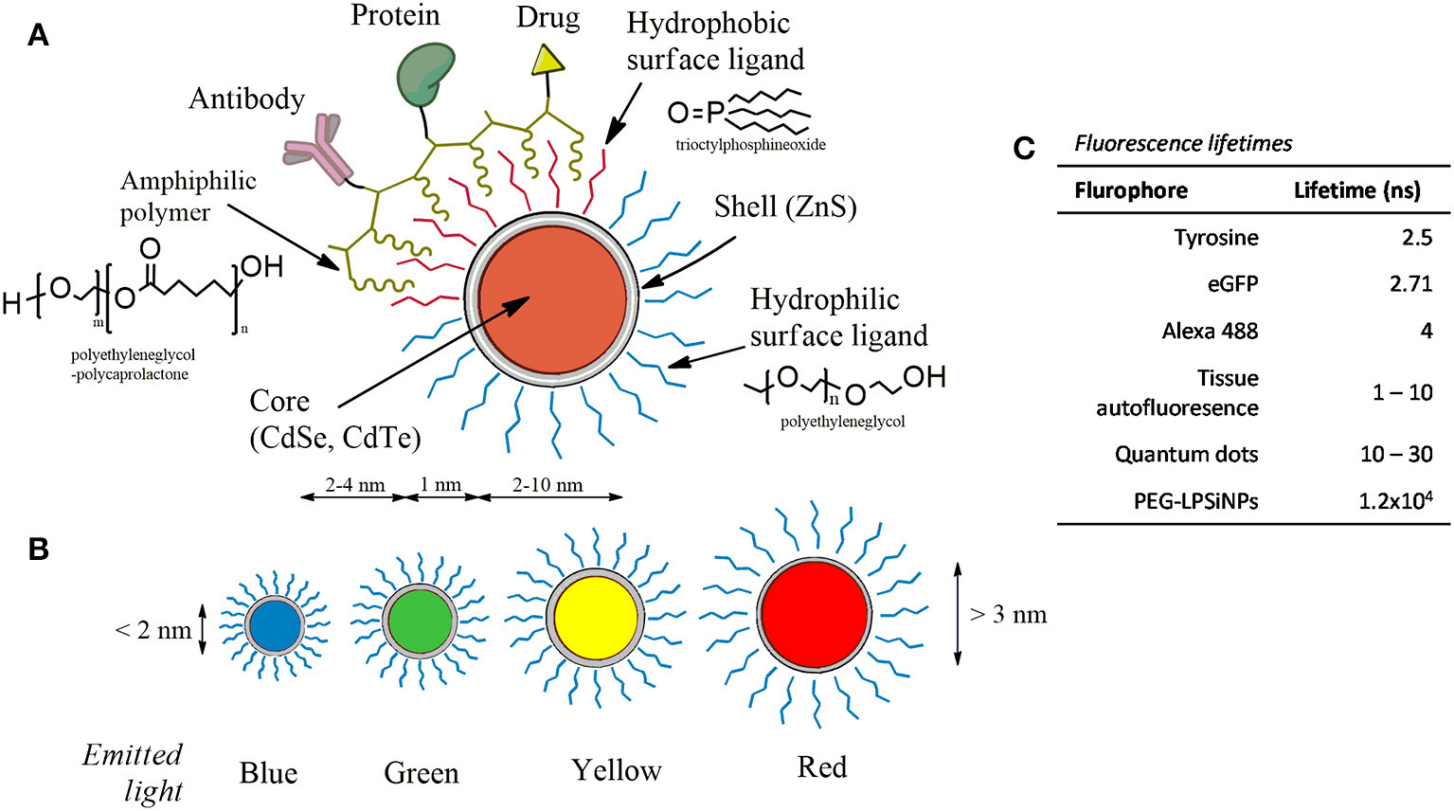
**The anatomy of quantum dots. (A)** QDs contain a semiconducting core-shell. The surface can be coated with hydrophilic, hydrophobic, and amphiphilic ligands (common coating molecules are shown) which can be further linked with proteins, drugs, antibodies, and other compounds. **(B)** The emission spectra of QDs can be tuned by adjusting the size. **(C)** Fluorescence lifetimes of QD in comparison with other flurophores (Berezin and Achilefu, 2010; Gu et al., 2013).

Among different nanotechnological products, quantum dots (QDs) attracted special attention because of their unique physicochemical and optical properties, some of which supersede certain qualities of fluorescent organic probes (Mattoussi and Cheon, 2009; Howes et al., 2014; Breger et al., 2015; Moloney et al., 2015; Silvi and Credi, 2015; Wegner and Hildebrandt, 2015). These properties include high fluorescent quantum yield, size-tunable emission and a broad absorption spectrum, ranging from ultraviolet to infrared wavelengths. QDs are composed of a semiconductor core (e.g., cadmium selenide (CdSe), cadmium telluride (CdTe), and are often capped with a shell [e.g., zinc sulfide (ZnS)] to improve core stability. There are also silica (Si)-based QDs which are highly stable and have long lifetime (Cooper et al., 2009). Figure 2 shows the anatomy of the QDs and a comparison between their lifetimes with those of fluorescent dyes and cell autofluorescence. In addition, the narrow emission spectra and broad excitation spectra allow for simultaneous detection of several different analytes *in vitro* and *in vivo*.

The exceptional photostability of QDs, relative to organic fluorescent dyes, makes them more suitable for biomedical labeling and imaging applications, particularly for longitudinal (repeated) imaging (Lidke et al., 2004; Cui et al., 2007; Rajan et al., 2008; Fichter et al., 2010; Vermehren-Schmaedick et al., 2014; Kovtun et al., 2015; Vu et al., 2015). For example, after binding to their receptor, TrkB, the internalization and intracellular trafficking of QD-BDNF (quantum dots with brain-derived neurotrophic factor ligand) was visualized. Generally, 2-h incubation with 1 nM QD-BDNF in the axon compartment led to 5–20 QD-BDNF-containing endosomes being transported in a 60 mm long axonal segment. It took ~40 min for the first QD-BDNF to reach the cell body and the accumulation of QD-BDNF in the cell body was observed within 2 or 3 h. This method can be used to address whether peripheral neurons in diabetic animals have impaired axonal transport (Xie et al., 2012; Zhao et al., 2014). Nerve growth factor (NGF), an important trophic factor for the survival and function of peripheral sympathetic and sensory neurons, has also been studied by employing QDs (Cui et al., 2007; Echarte et al., 2007). Results from these studies showed that NGF is retrogradely transported in axons of the rat dorsal root ganglia. Neural and other cells use tunneling nanotubes for intercellular communication ranging from electrical signaling to the transfer of organelles (Wang et al., 2012; Austefjord et al., 2014; Tosi et al., 2014; Wang and Gerdes, 2015). Tunneling nanotubes mediated transport of functional mitochondria can reduce the impact of ultraviolet light-induced insults. QDs can also be transported through tunneling nanotubes as shown by Zhang's group. Their studies show that QDs move bi-directionally with a mean velocity of 1.23 um/s (He et al., 2010). The velocity of the QD movement along the nanotube varies from 4.27 um/s to 0.054 um/s (mean velocity = 1.23 ± 0.01 um/s). Although most of the QDs reversed their directions of movement every few seconds, more than 80% of the QDS moved along the nanotube toward one of the connected cells. The likely microtubule associated motors mediating bidirectional transport of QDs are kinesins and dyneins.

One concern in using QDs with a Cd-based semiconductive core as sensors for live imaging is the potential cytotoxicity of Cd^2+^. To mitigate this concern, we investigated other types of QDs as potential candidates for sensors to probe neural cells. We showed that Indium-gallium phosphate/ZnS QDs (InGaP-QDs), core sizes of 5.0 nm and a fluorescence emission maximum at 680 nm, have low toxicity when tested in primary nerve cell cultures and in PC12 cells (Behrendt et al., 2009); indicating that these QDs are promising candidates for live cell and *ex vivo* imaging in the far red light spectrum. The strong signal that we observed from the internalized InGaP/ZnS QDs suggests that these nanoparticles aggregate in the cytoplasm, but not in the nucleus. In primary neural cultures enriched with astrocytes and glia, InGaP/ZnS QDs were internalized most avidly in microglia, followed by astrocytes, and were barely detectable in neurons. Quantitative analyses of internalized InGaP/ZnS QDs at the organellar level indicated that these NPs were mainly present in lysosomes but not in mitochondria (Behrendt et al., 2009).

Interestingly, we found that the subcellular distribution of InGaP/ZnS QDs is altered by oleic acid, a common ingredient of our daily diet (Behrendt et al., 2009). This finding suggests that changes in membrane structures by fatty acids (endogenous or exogenous) modulate the uptake and distribution of nanostructures in neural cells.

### PEBBLEs

Kopelman's team developed an interesting array of NP-based sensors called PEBBLEs (photonic explorer for biomedical use with biologically localized embedding; Sasaki et al., 1996; Clark et al., 1998; Lee et al., 2009). PEBBLEs are 1-1000 nm diameter nanoparticles that include both fluorescent analyte-sensitive *indicator* dyes and analyte-insensitive *reference* dyes (Lee and Kopelman, 2012b). As such, these sensors allow for ratiometric, reversible measurements and they are protected from interaction with the cellular environment. Two types of PEBBLEs are distinguished. Type 1 PEBBLE uses a single sensing entity, serving as both analyte recognizer and signal transducer, while in Type 2 PEBBLE the analyte recognizer and optical transducer are distinct. PEBBLEs have been developed to measure a number of physiologically relevant parameters, including ion concentrations (protons, calcium, copper, iron, magnesium, potassium, sodium, lead, zinc, chloride), small molecules (oxygen, singlet oxygen, peroxyl radical, hydrogen peroxide), enzymatic intracellular processes (apoptosis), and physical properties (temperature, electric field; Lee and Kopelman, 2012a). PEBBLEs have been used as sensors for intracellular pH and calcium concentration measurements in neural cells (Clark et al., 1999).

A good example of Zn ion sensor constructed as a PEBBLE (Type 2) is based on CdSe/ZnS QDs covalently linked with three different azamacrocycles, non-fluorescent Zn^2+^ ligands: TACN (1,4,7-triazacyclononane), cyclen (1,4,7,10-tetraazacyclododecane), and cyclam (1,4,8,11-tetraazacyclotetradecane; Ruedas-Rama and Hall, 2008). As the surface-conjugated azamacrocycles disrupt the radiative recombination process of the QDs, the QDs' fluorescence is quenched. The binding of Zn^2+^ with the azamacrocycles switches on the QD emission, resulting in an increase in fluorescence intensity. Three zinc ion sensors based on CdSe-ZnS core-shell QDs showed a very good linearity in the range 5–500 μM, with detection limits lower than 2.4 μM and relative standard deviation ~3%.

Although promising, one limitation of such zinc sensors is that interference from autofluorescence decreases their sensitivity. One-way to improve the intracellular sensitivity of the PEBBLEs is to avoid interference from cellular autofluorescence by using near infrared (NIR) fluorescent probes/reporters, two-photon excitation, and “MOON (modulated optical nanoprobe)” type PEBBLEs (Lee et al., 2009). MOONs are microscopic photonic probes that look like moons; one side appears bright and reports on the local microenvironment whereas the other side is dark. The MOONs rotate in response to thermal or magnetic fields (MagMOONs; Anker et al., 2005). The MOONs allow for sensitive chemical analyses where signal to background ratio can reach up to 4000-fold. Magneto-fluorescent MOONs have been more recently developed by Bawendi's group (Chen et al., 2014a). Their studies show that after surface PEGylation, these fluorescent “super” nanoparticles can be magnetically manipulated inside living cells. PEGylation is the covalent conjugation of poly-ethylene glycol to polymers and drug molecules. PEGylation prolongs the circulation half-life of drugs, reduces the immunogenicity of molecules, and stabilizes nanoparticles (Ginn et al., 2014; Kolate et al., 2014).

### Carbon nanomaterials (CNM)

Carbon nanomaterials have emerged as alternatives to QDs, molecular sensors, and bioengineered sensors (see Table 1). Carbon nanomaterials have unique electronic, magnetic and optical properties (Ding et al., 2014; Baptista et al., 2015; Wen et al., 2015; Zheng et al., 2015). In addition, carbon nanostructures such as graphenes, C-dots and nanodiamonds have relatively low toxicity, although there are some safety concerns regarding these materials (Bayat et al., 2015; Boyles et al., 2015; El-Sayed et al., 2015; Himaja et al., 2015; Jeannet et al., 2015; Lim et al., 2015; Misra et al., 2015; Pierrat et al., 2015; Qin et al., 2015b). To achieve the full potential of these structures as bionanosensors, further improvement is necessary. In particular, low sensitivity is a significant limitation (for example, detection of dopamine by graphene-based sensors). On the other hand, nanotubes seem to be useful tools for detecting DNA- drug interactions and could eventually become useful diagnostic aids in oncology (Health Quality, 2006). Developments in carbon nanomaterial based sensors were reviewed (Baptista et al., 2015).

An interesting example of a simple construct employing C-dots was used as a neuroanatomical retrograde tracer (Zhou et al., 2015). Cholera toxin B–carbon dot conjugates were taken up and retrogradely transported both in differentiated pheochromocytoma cells (PC12) and *in vivo* in Balb-c mice. Results from these studies suggested that cholera toxinB-modified C-dots were mainly taken up by dorsal root ganglia at the lumbar level L3–L5. Due to their superior properties (e.g., low toxicity, high signal intensity) over the Cd-containing QDs, C-dots are promising as multifunctional nanodevices for simultaneous multiple imaging modalities, drug delivery and sensing. However, currently available C-dot-based nanodevices have not yet reached this level of sophistication (Ding et al., 2014).

New fluorescent nanodiamonds are attracting considerable interest in the design of biological nanosensors due to their high resistance to photobleaching and low toxicity (Yu et al., 2005; Montalti et al., 2015). Compared to QDs and typical organic probes, nanodiamonds have high quantum yield and lifetime, although a limited tunability of emission spectrum and a larger emission width (Schirhagl et al., 2014). When tested in dorsal root ganglia cultures, nanodiamonds were highly fluorescent, but unfortunately, they also reduced growth cone extensions and neurite outgrowth (Huang et al., 2014). Similarly, when single-walled carbon nanotubes were tested in dissociated cultures of the peripheral nerves (Belyanskaya et al., 2009), they were found to be particularly toxic to Schwann cells, although less so for neurons. Current information on carbon nanodiamonds and carbon onions suggests that these morphologies may be better suited for nanosensors than carbon nanotubes due to their lower toxicity (Ding et al., 2005; Schrand et al., 2007). The morphology of carbon-based NP plays a role in internalization and intracellular trafficking (Chu et al., 2015). Importantly, neither prickly nanodiamonds nor morphologically similar Au-nanourchins are markedly cytotoxic (Hutter et al., 2010). Thus, both of these NP morphologies might be used for nanosensors to detect changes in cytoplasmic proteins under physiological and pathological conditions. Comparisons between different studies using such nanomaterials are difficult because experimental conditions including cell types, method of carbon nanomaterial preparation, and duration of exposure were different.

In summary, carbon-based nanosensors, particularly nanodiamonds and carbon onions seem to be promising nanomaterials for the construction of sensors to investigate neural cells under physiological and pathological conditions, once they are converted into fully biocompatible “biophils.”

### AuNP

Gold nanoparticles (AuNP) have several unique properties that make them attractive as components in biological nanosensors (Dykman and Khlebtsov, 2012; Jin, 2014). These include: a high absorption coefficient (e.g., 40 nm gold nanospheres show an absorption cross-section 5 orders of magnitude higher than organic dyes), scattering flux (e.g., 80 nm gold nanospheres scatter light 5 orders of magnitude compared to some fluorescent dyes), luminescence and conductivity, the ability to enhance electromagnetic fields, quench (or enhance) fluorescence, and catalyze reactions. As such, these nanosensors are the most common (e.g., see Table 1).

Many shapes and various surface modifying molecules have been exploited for AuNP-based nanosensor construction (Vo-Dinh et al., 2013; Liu et al., 2015; Qin et al., 2015a; Zhang et al., 2015c). Gold nanorods, nanoshells and nanourchins are particularly attractive tools for *in vivo* applications, since their optical resonance lies in the near-infrared spectral window, away from the region of biomolecular excitation transitions, which precludes photochemical damage and allows for deeper penetration of light in living systems. It is important to note, however, that cellular internalization of NPs depends on the shape, size and surface properties of the NPs just as much as on the cell type. For example, when spherical, rod and urchin GNPs were added to primary hippocampal neurons and microglial cells, Au nanourchins were found to be taken up preferentially by microglia, while only Au nanorods were internalized by neurons (Hutter et al., 2010).

Characterization of AuNPs is essential for any sensor construction (Leifert et al., 2013; Conde et al., 2014). The most common techniques to determine the particle size and its distribution (core and shell) and the successful ligand attachment are electron microscopy, absorption spectroscopy, dynamic light scattering, asymmetric flow-field flow fractionation (Moquin et al., 2013b, 2015), and zeta potential measurements. Elemental analysis, thermogravimetric analysis/differential scanning calorimetry, nuclear magnetic resonance spectroscopy, infrared spectroscopy and X-ray photoelectron spectroscopy can provide some quantitative information about the ligand shell composition and functionality (Leifert et al., 2013).

To generate a sensor, the ligand is covalently attached to the AuNP surface by a dative metal-thiol bond (Howes et al., 2014). The bond dissociation energy for AuNP—S bonds is ~40–50 kcal/mol, approximately half of that for a typical C—C or C—H bond. In contrast, AuNP—N (~8 kcal/mol) and AuNP—COO—~2 kcal/mol) dissociation energies are considerably weaker, comparable in strength to a hydrogen bond, and can easily be displaced (Muddineti et al., 2015).

AuNPs can function in conjunction with many traditional biological probes such as antibodies, nucleic acids, ligands and receptors, and are used for a highly sensitive and selective detection of various biomarkers (see Table 1). Some of these assays have already been commercialized (Nanosphere, Merck, BBInternational, etc.). Measurements revealing changes in the cellular environment components and enzymatic activities are important in the exploration of physiological and pathological processes such as neuroinflammation. Enzymes often activated in neural cells in inflammation are caspase-1, cyclooxygenases and matrix metalloproteinases (MMPs), among others. Nanosensors for MMPs based on AuNP and their brief description are included in Table 1 and are discussed in Section Nanosensors for Proteases and Aromatase.

### Bioengineered sensors

With genetically encoded fluorescent proteins, one can study dynamic changes in intracellular processes in live cells, and quantify the expressions of proteins which are difficult to measure by other means (Enterina et al., 2015). Fluorescent protein based biosensors are useful tools for the study of signaling processes in neurons (Shen et al., 2015). This approach was recognized as an invaluable contribution to biological investigations including those in neuroscience by the Nobel Prize in chemistry granted to Osamu Shimomura, Martin Chalfie and Roger Y. Tsien. Genetically encoded proteins are either used to label and image proteins of interest without fundamental changes in their properties (imaging tools) or to “sense” complex biochemical processes in living cells (Frommer et al., 2009; Oldach and Zhang, 2014). Some examples of genetically engineered sensors and their properties that can be exploited in investigating neural cells under physiological and pathological conditions are shown (Table 3).

**Table 3:** **Examples of genetically engineered biosensors for the detection of ions and enzymes in neural cells**.

**Analyte**	**Sensor**	**Measured in**	**Detection principle**	**Reference**
Glucose	FRET FLII12PGLU-700uΔ6 glucose nanosensors (Ratiometric)	Dissociated rat astrocytes	Recognition based. Binding of glucose to the glucose/galactose binding domain (Mg1B) causes a conformational shift that increases FRET efficiency between CFP and YFP. The transcript was chemically transfected into cells and constitutively expressed.	Prebil et al., 2011
Lactate	Laconic (Ratiometric)	*In vivo*, cortical slices, dissociated cultures from mice	Recognition based. Binding of lactate to LldR causes a conformation change that decreases FRET efficiency between mTFP and venus. The transcript was chemically transfected into dissociated cultures, and introduced into slice cultures using n adeno-viral vector.	Sotelo-Hitschfeld et al., 2015
Calcium	FRET-based Ca^2+^ sensor (TN-XXL) (Ratiometric)	*In vivo* (mice brain)	Recognition based. Binding of calcium to troponin C (TnC) causes a conformational shift that increases FRET efficiency between CFP and cpCitrine. The transcript contained a Thy1 promoter and was constitutively expressed *in vivo* in neurons.	Siffrin et al., 2015
Calcium	Twitch (Ratiometric)	Neurons *in vivo* (rat visual cortex)	Recognition based. Binding of calcium to TnC causes a conformational shift that increases FRET efficiency between CFP and cpCitrine. The transcript was expressed in mouse primary visual cortex (V1) using an adeno-viral vector.	Thestrup et al., 2014
Calcium	sPA-GCaMP6 (Single wavelength)	Rat hippocampal slices, *in vivo* fruit fly, zebrafish	Recognition based. Illumination at 405 nm activates the sensor while calcium binding to GCaMP increases fluorescence at 510 nm. The transcript was expressed in primary rat hippocampal slices, fruit fly, and zebrafish through various means.	Berlin et al., 2015
Caspase	Fluorescent protein exchange (FPX) biosensor (Ratiometric)	Dissociated rat neural cells	Enzymatic cleavage. Dimerization-dependent fluorescent proteins RA and B are linked by a caspase substrate peptide. Caspase activity in the cytosol lead to a decrease in red fluorescence due to separation of RA and B. Subsequent nuclear translocation of B lead to the association of GA and B, which increases green fluorescence in the nucleus. The protein sensor was injected into the cytosol of neurons using a microinjector.	Ding et al., 2015
Chloride	Clomeleon (Ratiometric)	Dissociated rat neurons	Change in fluorescence intensity. YFP fluorescence is dependent on chloride levels while CFP fluorescence is not. Increasing Cl- decreases YFP fluorescence resulting in an increase in CFP/YFP fluorescence ratio. The transcript was expressed in primary dissociated cultures by using electroporation.	Kuner and Augustine, 2000

Bioengineered sensors presented in Table 3 are useful sensors for the detection of cell metabolites, ions, and enzymes. Similarly, bioengineered sensors provide the means of detection for different kinases and their participation in spatially restricted areas and their function in macro-compartments (i.e., dendritic spines) or micro-compartments (i.e., AKAP signalosomes; Willoughby et al., 2010; Yasuda, 2012). They can be used to determine enzymatic activities in real-time and in a cell specific manner. Transgenic expression of reactive oxygen species (ROS) generating proteins (RGPs) as fusions to native proteins allow for exertion of spatial and temporal control over ROS production and ROS signaling (Wojtovich and Foster, 2014).

## Nanosensors for proteases and aromatase

### AuNP-based sensors for MMPs

Matrix metalloproteinases (MMPs) are pleiotropic endopeptidases involved in a variety of neurodegenerative processes including neuroinflammation (Sonderegger and Matsumoto-Miyai, 2014; Yamamoto et al., 2015). They interact with a large number of substrates (Malemud, 2006). Most of the MMPs are synthesized as inactive latent enzymes. Conversion to the active enzyme is generally mediated by activator systems that include plasminogen activator or the pro-hormone convertase, furin. MMPs are active at physiological pH and they catalyze the normal turnover of extracellular matrix (ECM) macromolecules. The endogenous inhibitor of MMPs (TIMP-2) is constitutively expressed in microglia and markedly inhibited by proinflammogen lipopolysaccharide (LPS) treatment (Lee and Kim, 2014). Knockdown and overexpression experiments in microglial cell line BV2 suggest that endogenously expressed TIMP-2 plays an anti-inflammatory role. Overexpression or knockdown of TIMP-2 in BV2 cells leads to reciprocal expression and release of inflammatory biomarkers after LPS treatment. Overexpression of TIMP-2 in BV2 cells doubles the expression of the anti-inflammatory IL-10 and markedly decreases the expression of pro-inflammatory cytokines TNF-α, IL-1β, as well as nitric oxide. Conversely, siRNA knockdown of TIMP-2 in BV2 cells reduces the expression of IL-10 by ~ 30% and increases ROS, nitric oxide, and TNF-α. An overexpression of TIMP-2 suppressed microglial activation through inhibition of the activity of mitogen-activated protein kinases (MAPKs) and transcription factor NF-κB. The results from these studies indicated an enhancement of the activity of anti-inflammatory Nrf2 and cAMP-response element binding protein (CREB) transcription factors in microglia with overexpressed TIMP-2. Microglia activation contributes to the degradation of extracellular matrix proteins by increasing metalloproteinases activities (Lively and Schlichter, 2013). In the transgenic 5X FAD mouse model of Alzheimer's disease (AD) the expression of MMP-2, MMP-9, and MT1-MMP was upregulated concomitantly with the tissue inhibitor of MMPs-1 (TIMP-1) and several markers of inflammatory/glial response (Py et al., 2014). Data from these studies suggest a regulatory interplay between MMPs and the amyloid precursor protein (APP). The role of astrocytes and MMP-9 in synaptic dysfunction has been reviewed (Kamat et al., 2014).

In fluorescence-based assays, including these for MMPs, AuNPs are employed as acceptors, quenching the emission of donor chromophores. Because of their large absorption cross section, AuNPs have a superior quenching efficiency in a broad range of wavelengths compared to other organic quenchers. Therefore, they can be used for studies in which donor-acceptor distances are expected to extend beyond 10 nm, or studies in which multiple dyes need to be quenched. Since they have no defined dipole moment, energy transfer takes place for any orientation of the donor relative to the surface of the AuNPs. For instance, even if the distance between AuNPs and fluorescent dyes are as large as 22 nm, the quenching efficiency can be as high as 95% (Mayilo et al., 2009).

An example of one such assay detects MMP-7. MMP-7 is an extracellular protease that exerts a broad range of biological functions including important roles in synaptic plasticity (Sonderegger and Matsumoto-Miyai, 2014). In the assay for MMP-7 detection, carboxy AuNPs (5 nm in core diameter) are used as both quenchers and metal chelators, and are strongly associated with the hexahistidine regions of dye-tethered peptides in the presence of Ni(II) ions; this leads to fluorescence quenching of the dye by AuNPs. Upon adding MMP-7, the peptide is cleaved and the fluorescent intensity of the dye is efficiently recovered. The degree of dequenching is directly dependent on the MMP-7 concentration in a hyperbolic manner, ranging from as low as 10–1000 ng mL^−1^ (Park et al., 2012).

MMP-2 is a constitutive protein found in the normal brain cardiovascular systems and glioma (Lebel and Lepage, 2014; Mittal et al., 2014; Wang et al., 2014, 2015c; Ruan et al., 2015; Yang and Rosenberg, 2015). Experimental evidence suggests that MMP-2 may contribute to early brain enhancement of the cytokine interleukin-1 beta concentrations in transient ischemia thereby promoting cortical neuron damage (Amantea et al., 2014). MMP-2 measurements *in vivo* can be achieved by exploiting near-infrared-fluorescent dye functionalized gold nanoparticles. The fluorescent dye is quenched until the protease cleaves its link to the AuNP and releases it. Tumors with high protease activity can be visualized by the near infrared fluorescence signals from gold nanoparticle probes upon MMP-2 activation (Lee et al., 2008).

MMP-9 has been recognized as a regulator of dendritic spine morphology. Dendritic spines are dynamic structures that change their morphology in response to various stimuli (Stawarski et al., 2014b). Spine remodeling occurs in many degenerative disorders and MMP-9 seems to play a critical role. Stawarsky et al. developed a biosensor to measure MMP-9 activity in living cell (Stawarski et al., 2014a). This biosensor can be used to monitor the changes of MMP-9 activity and correlate them with plastic changes of dendritic spines. Considering the accessibility of AuNP sensor to the spines, it is likely that AuNP sensors would be useful in assessing MMP-9 activity and be correlated with spine morphology and function. Extracellular proteolytic cleavage at synapses is executed by a relatively small number of peptidases which have a limited set of target proteins. MMP-9 is one of the peptidases which plays critical roles in synaptic structure and function. MMP-9 expression is enhanced by reactive oxygen species (ROS; e.g., post injury, high glucose; Hsieh et al., 2014). MMP-9 might play a dual role in epilepsy (Michaluk and Kaczmarek, 2007). It is also highly expressed in microglia in response to inflammatory stimuli (Gottschall et al., 1995) and can induce neuronal cell death (Murase and McKay, 2012; Gao et al., 2015). Analyses of MMP-9 deficient mice showed a significant reduction in neuronal damage in the hippocampus after transient global cerebral ischemia (Lee et al., 2004), but in dendritic spines conflicting results (enlargement and thinning) were reported. These controversial issues, as well as the roles of metalloproteinase 2 and 9 in the development, plasticity and repair of the nervous system were reviewed (Verslegers et al., 2013).

MMPs increase the permeability of the blood–brain barrier as part of the neuroinflammatory response in hypoxia–ischemia, multiple sclerosis and infection (Rosenberg, 2009). Recent studies have also implicated MMPs in the chronic neurodegeneration associated with vascular cognitive impairment, Alzheimer's disease, and Parkinson's disease. Therefore, both the identity of the active MMPs and their cellular origin could determine whether disease pathogenesis or regeneration occurs. Thus, synthetic MMP inhibitors might be valuable for treating some CNS diseases (Rosenberg, 2009).

### Bioengineered sensors for MMPs

Only recently an MMP-9 sensor has been developed using mCherry fusion protein to quantify intracellular protein and secreted protein in the extracellular medium (Duellman et al., 2015). This microplate reader-based mCherry fluorescence detection method had a wide dynamic range of 4.5 orders of magnitude and a sensitivity that allowed detection of 1–2 fmol fusion protein. The rate of secretion was calculated from the linear region of data from 8 to 24 h post-transfection. Comparison with the Western blot protein detection method indicated greater linearity, wider dynamic range, and a similar low detection threshold for the microplate-based fluorescent detection assay of secreted fusion proteins.

### AuNP and QD-based sensors for caspases

Many neurological disorders are associated with inflammation (Cardoso et al., 2015; Crotti and Glass, 2015; De Felice and Lourenco, 2015; Franco and Fernández-Suárez, 2015; Freeman and Ting, 2015; Hayashi and Cortopassi, 2015; Hoogland et al., 2015; Maphis et al., 2015; Ward et al., 2015); consequently, enzymes implicated in inflammatory processes are likely good targets for therapeutic interventions (Py et al., 2014; Kaushal et al., 2015; Savard et al., 2015; Yang and Rosenberg, 2015; Wang et al., 2015a). The initiation of inflammatory responses involves the formation of cytosolic structures named “inflammasomes” (Martinon et al., 2002; Fang et al., 2015; Frank et al., 2015; Freeman and Ting, 2015; Guo et al., 2015; Szabo and Petrasek, 2015; Yang and Chiang, 2015). Inflammasomes are multiprotein complexes that enable the activation of pro-inflammatory caspases, mainly caspase-1 (Gross et al., 2011). Mechanism of caspase-1 and caspase-11 activity was reported (Kayagaki et al., 2015; Shi et al., 2015). Different caspases recognize different peptide sequences and cleave them. These caspase-specific peptides (substrates) can be incorporated in sensors as linkers between fluorogenic and/or quenching entities. As a consequence of enzymatic cleavage, there is a change in fluorescence intensity. Since the identification of sequences that are cleaved in various caspase substrates (McStay and Green, 2014; Kang et al., 2015; Parsons et al., 2015) and the development of synthetic substrates, these sensors became attractive to reveal enzyme-specific reactions in different cells, including neural cells, but mainly in lysed cells. Real-time measurement of caspase activity in live cells and animals is more challenging, but with the advancement of technology (particularly nanotechnology) it has become possible (Ai et al., 2008; Hutter and Maysinger, 2013; Moquin et al., 2013a). Examples of QD- based nanosensors for measuring enzymatic activities were reviewed (Hutter and Maysinger, 2013) and are given in Table 1.

**Figure 3:**
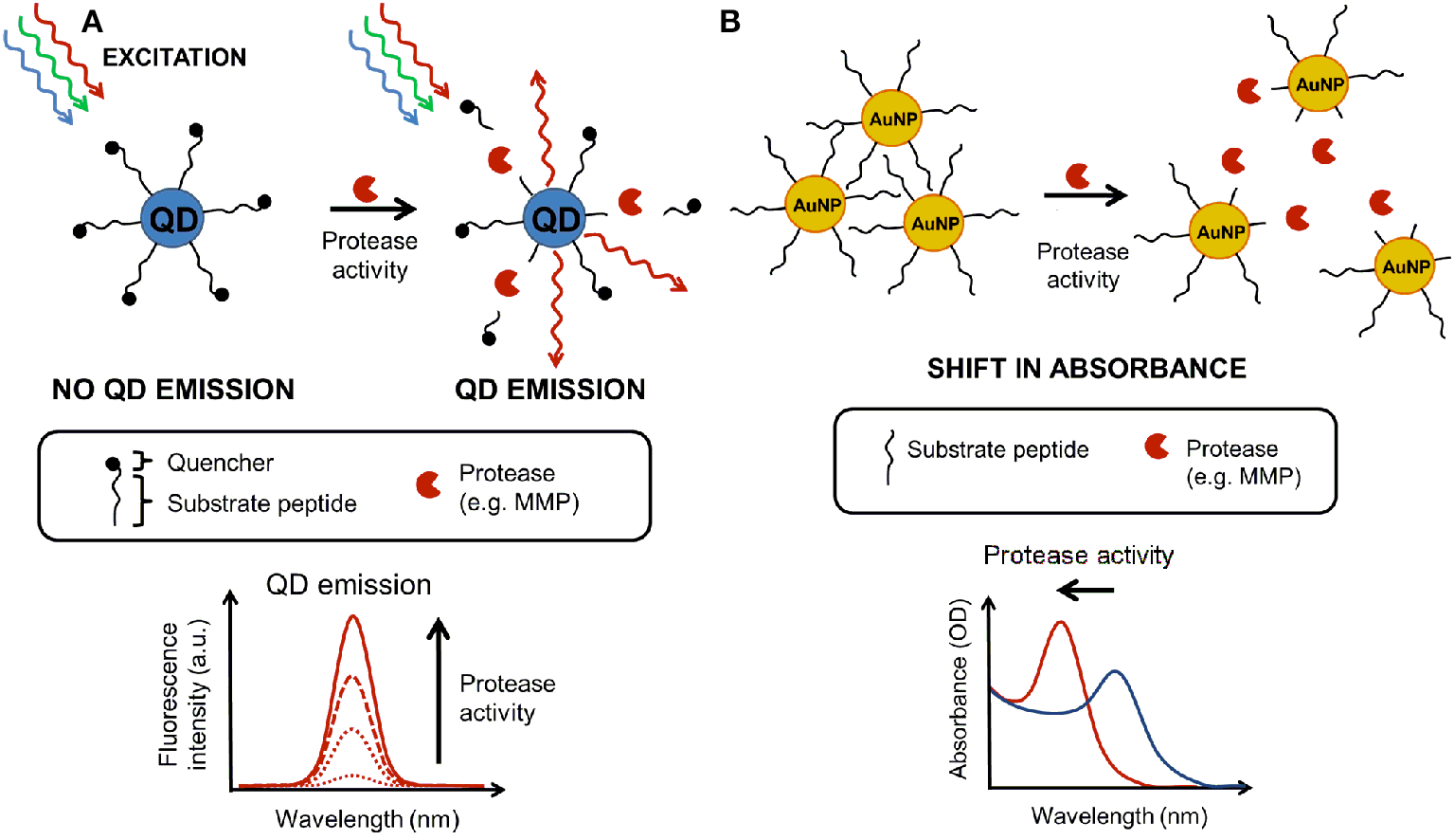
**Nanosensor measurements based on absorbance and fluorescence**. **(A)** Principle of a fluorescence-based nanosensor where a quantum dot (QD) is linked to quencher molecules through substrate linkers. An active protease cleaves off the link leading to dequenching (enhanced fluorescence proportional to protease activity). **(B)** The principle of an absorbance-based nanosensor using gold nanoparticles (AuNP) functionalized with cross-linked peptides [causing the aggregation of the AuNP which have an absorbance peak around 600–700 nm (OD)]. An active protease chews the substrate peptide, causing the disaggregation and a blue shift in the peak absorbance (500 nm).

The basic principles of enzymatic activity measurements using QDs and AuNP are changes in fluorescence intensities (due to the substrate cleavage) or shift in absorbance maximum (due to the change in aggregation status of nanoparticles), as illustrated (Figure 3).

**Figure 4:**
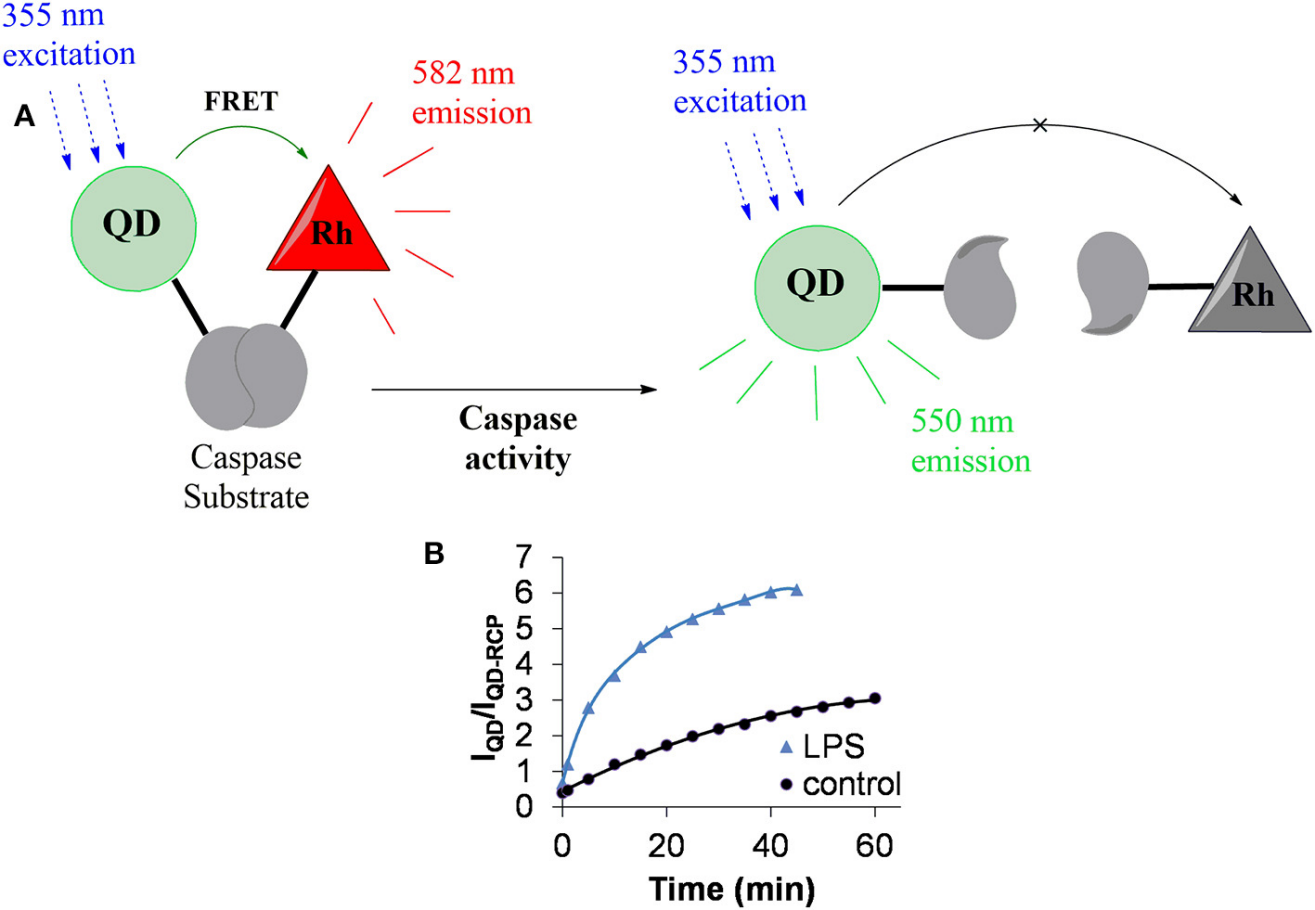
**QD-based nanosensor for the detection of caspase-1 activity. (A)** In the absence of caspase activity, FRET between the quantum dot (QD) and rhodamine (Rh) results in the latter's fluorescence emission at 582 nm (shown in red). Active caspase cleaves the substrate peptide linking the QD and rhodamine and FRET is abolished. This results in the loss of Rh fluorescence and the gain of QD emission (shown in green). **(B)** Representative measures of caspase-1 sensor activity using QD-based sensor. LPS induces caspase activation which leads to rapid cleavage of the peptide link between QD and Rh an increase in the fluorescence ratio of I_QD_ (550 nm)/I_QD-*RCP*_ (582 nm). RCP, Rhodamine conjugated peptide.

To investigate how microglia respond to proinflammatory stimuli, our laboratory has developed a QD-based assay for caspase-1. Caspase-1 activity in this assay is determined by ratiometric measurements of fluorescence signals based on FRET (Figure 4; Moquin et al., 2013a). An attractive feature of our assay is that we could follow caspase-1 activity over time, because the signals from QDs are more stable than conventional organic fluorophores. Our sensor is suitable for measuring changes in caspase-1 activity at the single cell level.

Aside from QD, AuNPs could also be used for caspase sensor construction. The SPR peak of AuNPs is very sensitive to their environment, shifting toward the longer wavelengths (red shift) and broadening significantly upon the aggregation of AuNPs, i.e., the absorption peak of dispersed particles changes toward longer wavelengths when the particles are aggregated. This color change is easily measured by conventional spectrometers. The color shift can also occur in the reverse direction: breaking the AuNP aggregates into individual particles causes a blue shift in the absorption spectrum of dispersed particles. The basic principle of AuNP-based colorimetric assays is that the extent of aggregation/separation is proportional to the absorption peak shift, and therefore the signal is quantifiable. By monitoring the ratio of the area under the surface plasmon peak of aggregated AuNPs (spanning from 490 to 540 nm) and the area under that of dispersed particles (550–700 nm), it is possible to obtain a ratiometric quantification of enzymatic activity. Although rarely used, the power of this approach has provided an extremely high detection limit of 90 zeptograms/mL(10^−21^ g/mL) for thermolysin (Laromaine et al., 2007). A similar approach could be used for sensors consisting of AuNPs assembled through protease cleavable peptides and dispersed in the presence of the active enzyme. Such a sensor could be easily applied to the measurement of enzymatic activities of various proteases, particularly those imbedded in plasma membranes with their active site exposed to extracellular environment.

### Bioengineered sensors for caspases

**Figure 5:**
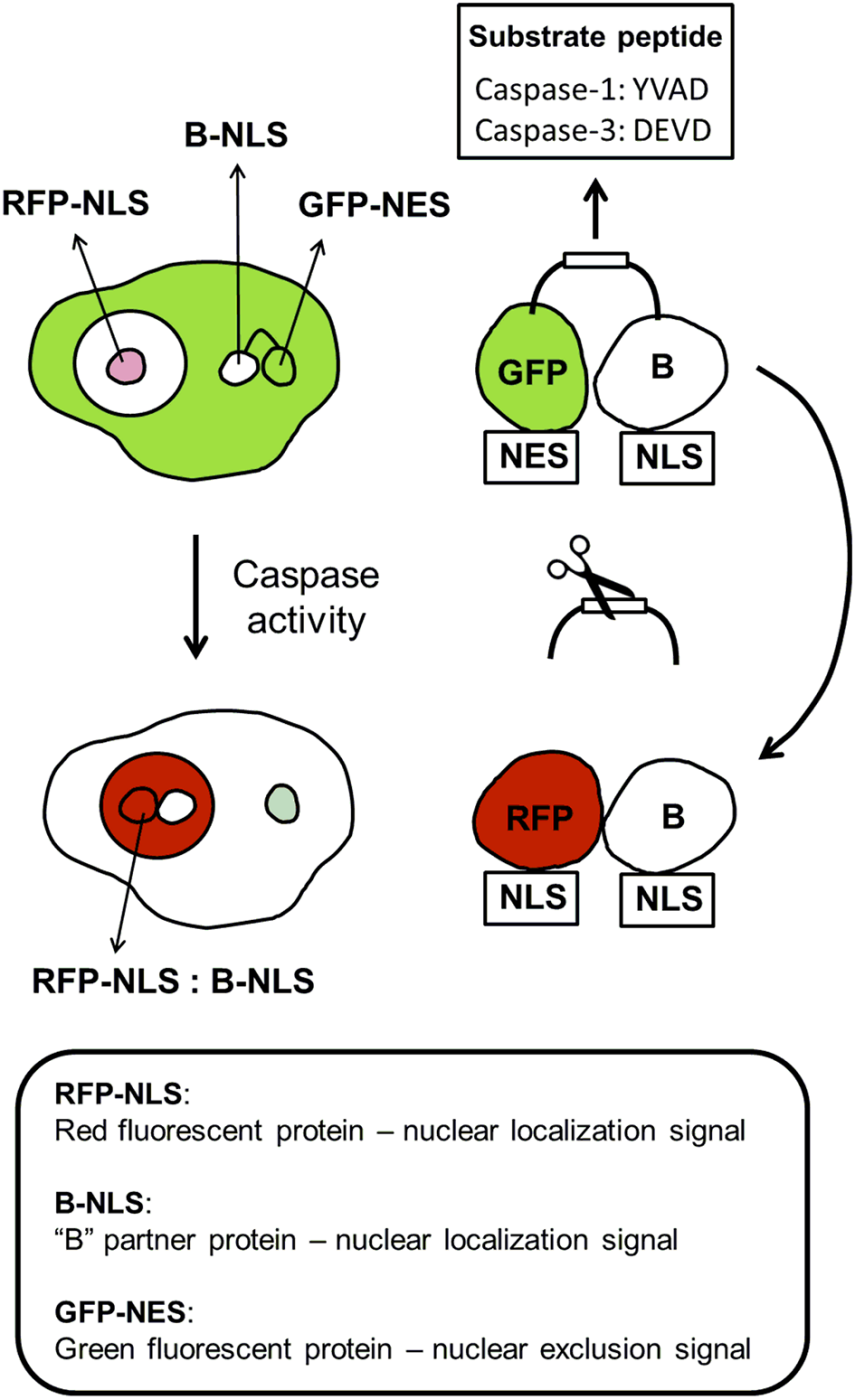
**Components of protein dimerization-based sensors for caspase-1 and caspase-3**. The dimerization-based caspase biosensor relies on the expression of two plasmid-encoded, dimerization-dependent fluorescent proteins: (1) a green fluorescent protein tagged with a nuclear exclusion signal (GFP-NES) and linked to a partner protein “B” tagged with a nuclear localization signal (B-NLS) by a caspase substrate peptide (Caspase-1: YVAD; Caspase-3: DEVD), and (2) a red fluorescent protein tagged with a nuclear localization sequence (RFP-NLS). Caspase activation causes the substrate cleavage. B-NLS translocates to the nucleus, where it binds to RFP-NLS. The binding of B-NLS, which itself is non-fluorescent, to GFP-NES or RFP-NLS increases the fluorescence of either dimerization-dependent fluorescent proteins many folds. Thus, cells expressing the caspase biosensor show green fluorescence in the cytoplasm when caspase activity is low and red fluorescence in the nucleus with increased caspase activity.

In addition to nanoparticle-based sensor for caspases, there are several bioengineered sensors for this class of proteases (Table 3 and Figure 5). The suitability of a FRET-based sensor for caspase-3 was demonstrated in 3D-cultures using breast cancer cells, but similar construct could be used for the determination of caspase 3-activity in neural cells (Anand et al., 2015). Caspase-3 has been implicated not only in cell death but also in synaptic failures in the absence of cell death (D'Amelio et al., 2011). The molecular mechanisms underlying synaptic failure are still incompletely understood, but studies by D'Amelio et al. identified a caspase-3-dependent mechanism that drives synaptic failure and contributes to cognitive dysfunction in Alzheimer's disease mouse model and possibly in humans (D'Amelio et al., 2011).

Multiple roles of caspase-1 in neuroinflammation have been reported in several animal models (Alfonso-Loeches et al., 2014; Freeman and Ting, 2015). For example, CNS human neurons express functional NLRP1 inflammasomes, which activate caspase-1 and subsequently caspase-6. Studies by LeBlanc (Kaushal et al., 2015) reveal a fundamental mechanism linking intraneuronal inflammasome activation to caspase-1-generated interleukin-1-β-mediated neuroinflammation and caspase-6-mediated axonal degeneration. The basic principle of protein dimerization-based bioengineered sensors for caspase-1 and caspase-3 is illustrated (Figure 5).

Using sensors for caspase-1 and caspase-3 (Figure 5), we showed that human cells exposed to lipopolysaccharide markedly activated caspase-1, but not caspase-3. In turn, caspase-3, but not caspase-1, was activated when cells were exposed to staurosporine (Ding et al., 2015). These bioengineered sensors for caspase-1 and 3 can be a great help assessing the microenvironment-modifying agents and antineoplastic agents in glioblastoma and also in neurodegenerating CNS.

### Aromatase function and measurements

**Figure 6:**
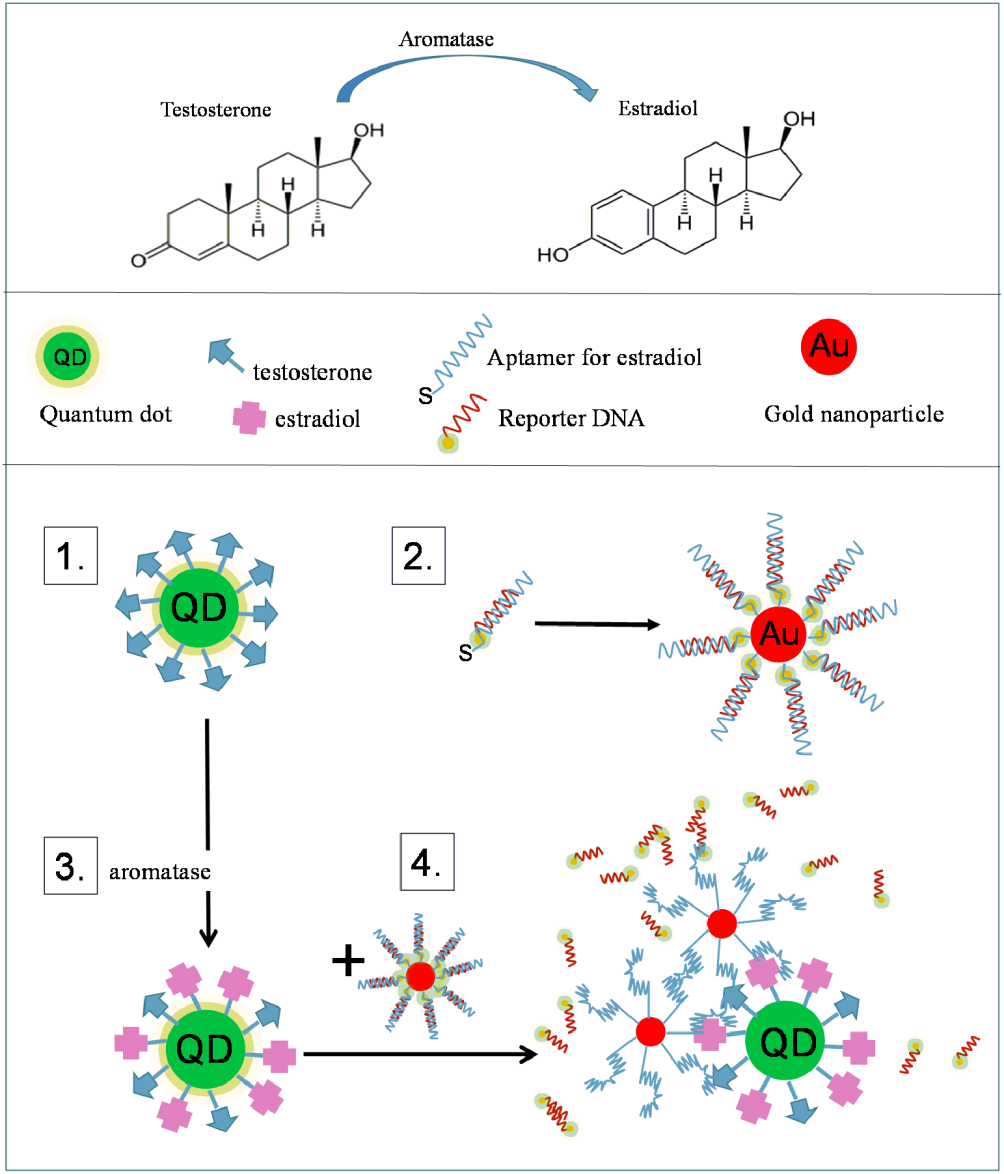
**A schematic of the aromatase AuNP-based sensor**. Aromatase converts testosterone to estradiol. **(1)** The substrate of aromatase, testosterone is covalently conjugated to the surface of quantum dots, QDs (“QD-testosterone”). **(2)** Thiol-terminated estradiol-binding aptamers are allowed to form a complex with their fluorophore-labeled complementary DNA strand (reporter DNA). This complex is attached to the surface of AuNPs through the thiol groups of the aptamer (“AuNP-aptamer”). The fluorophore is quenched by the AuNP. **(3)** In the presence of aromatase some of the QD conjugated testosterone is converted to estradiol. **(4)** Upon addition of AuNP-aptamers, the fluorescently labeled reporter DNA is released and the aptamer binds to the estradiol. The fluorescence of QDs is quenched by the close proximity of AuNP, while the fluorescence of the released reporter DNA is restored. In addition, the aggregation of AuNPs and QDs results in a very distinct change in the surface plasmon absorption spectrum of AuNPs. The triple detection system (QDs, fluorophore and AuNP) allows for monitoring conversion of testosterone to estradiol separately (by QD quenching, fluorophore turn-on, NP aggregation).

Neural-active steroids play a critical role in the development of the central nervous system and in the maintenance of functional circuitries (Melcangi et al., 2011; Remage-Healey et al., 2011; Arevalo et al., 2015; Frankfurt and Luine, 2015; Hojo et al., 2015; Krentzel and Remage-Healey, 2015). Aromatase is the key enzyme which transforms testosterone into estradiol (Yague et al., 2010). The measurements of aromatase enzymatic activity are mainly indirect and based on radioactive measurements of tritiated water. There are currently no ways to measure aromatase activity in neural cells directly and non-invasively. Some neurological disorders, particularly in postmenopausal women are ascribed to inadequate estrogen concentrations in certain brain structures, e.g., hippocampus (Danilovich et al., 2003; Markham et al., 2005; Spence and Voskuhl, 2012; Daniel, 2013). Aromatase inhibition by letrozole is used in breast cancer therapy and there are reports that such therapeutic interventions can cause memory impairments in certain female populations; however, it is not clear what makes them more vulnerable to letrozole treatment (Zhou et al., 2007; Chang et al., 2013; Turnbull et al., 2015; Vierk et al., 2015). In this context, we have shown that in organotypic hippocampal cultures treated with letrozole, post-synaptic dendritic spines are reduced in number, resulting in dysfunctional neural circuitry (Chang et al., 2013). We propose a FRET-based assay for the measurement of aromatase enzymatic activity by using nanoparticles (Figure 6).

In theory, AuNPs and QDs can be combined in a FRET-based assay. A good example is the design to detect the activity of aromatase (Figure 6). In this strategy, the substrate of aromatase, testosterone is covalently conjugated to the surface of quantum dots (“QD-testosterone”). AuNPs are modified with a complex of thiol-terminated estradiol-binding aptamers (“AuNP-aptamer”) and their fluorophore-labeled complementary DNA strand (reporter DNA; Alsager et al., 2015). In the presence of aromatase some of the QD-conjugated testosterone is converted to estradiol; then, upon addition of AuNP-aptamers, the fluorescently labeled reporter DNA is released and the aptamer binds to the estradiol, bringing the AuNPs into a close proximity of QDs. The fluorescence of QDs is quenched by the AuNP, while the fluorescence of the released reporter DNA is restored. In addition, the aggregation of AuNPs and QDs results in a very distinct change in the surface plasmon absorption spectrum of AuNPs. This elegant triple detection system (QDs, fluorophore and AuNP) allows for monitoring of the testosterone to estradiol conversion by three parallel ways (by QD quenching, fluorophore turn-on, NP aggregation). Such an assay is not yet available but it would be very useful in experiments and clinical studies addressing questions related to the role of aromatase in neurological disorders.

In summary, we have identified a number of nanoparticle-based sensors for enzymes relevant in the nervous system and we have highlighted both their advantages and limitations. The most common sensors are those for caspases and MMPs. These enzymes are particularly relevant because of their roles in synaptic plasticity and neurodegenerative disorders. Being extracellular enzymes, MMPs are relatively more easily accessible by nanomaterials than intracellular enzymes such as caspases. MMPs, acting as extracellular matrix and spine “sculptors,” play essential roles in neural cell functions; therefore, further in-depth studies are warranted to unravel the intricate roles of MMPs, particularly their potential beneficial contributions in post-injury repair of the nervous system. Nanosensors that allow simple and reproducible measurements of MMPs enzymatic activities could facilitate these investigations. These assays would be of value for designing new therapeutic interventions in neurological disorders where abnormal regulation of these proteases contribute to neural malfunction and death. Analyses of various MMP substrates incorporated into nanoparticle-based sensors could certainly help delineate preferable specific substrates in neural cells and downstream pathways leading to beneficial or detrimental MMP-mediated functions. Finally, understanding MMP-mediated proteolysis in neural cells would go far beyond these cell types.

## Sensors for ions and cell metabolites

In this section, we will highlight several examples that incorporate genetically engineered or NP-based sensors. We will focus mainly on pH, O_2_ and Ca^2+^ measurements using nanosensors.

### Bioengineered sensors for the detection of calcium ions

Ever since Ringer's serendipitous observation that Ca^2+^ caused the contractions of isolated hearts, physiological roles of this ion were intensively investigated (Ringer, 1883). The extracellular calcium concentrations are high (about 1 mM), whereas intracellular pools are low (100 nM). The most important calcium stores are the endoplasmic reticulum and mitochondria. Special temporal patterns of calcium are regulated by pumps, channels, and buffering proteins. Regulation of calcium signaling and the role of mitochondria in its regulation contributing to cell metabolism, and cell survival has been reviewed (Rizzuto et al., 2012).

In neurons calcium is in constant flux to facilitate neurotransmission and even modest alterations in calcium signaling can cause cellular stress and negatively impact cell function (Kawamoto et al., 2012). For instance, in Alzheimer's disease, neuronal calcium disturbances and abnormally regulated calcium signaling proteins are speculated to play a major role in causing mitochondrial dysfunctions (Supnet and Bezprozvanny, 2010). The variety of cellular responses to calcium signaling depends on the duration, subcellular location, and the amplitude of the change. Calcium signaling can occur in microdomains or macrodomains. Local increases in calcium concentration in neuronal presynaptic terminals triggers neurotransmitter release (Neher and Sakaba, 2008). Global changes such as elevated calcium levels can trigger autophagy, apoptosis amongst other processes in neural cells (Matute et al., 2006; Wojda et al., 2008; Brini et al., 2014). Activation of different signaling pathways depends on the extent of calcium influx; the strength of NMDA-dependent calcium influx determines whether long-term potentiation or depression occurs in hippocampal neurons (Lüscher and Malenka, 2012).

Currently, synthetic calcium flurophores such as Fluo-3 or Fura Red (see Table 2) are popular tools for quantifying calcium concentrations since they allow short term spatiotemporal monitoring of Ca^2+^ concentration. However, these synthetic dyes require loading, have limitations related to variable dye entry and leakage from cells and thus are not suitable for long-term or *in vivo* monitoring of calcium levels (Kantner et al., 2015; Thomas and Oliver, 2015). Furthermore, these dyes are not tissue or organelle specific. Genetically encoded calcium indicators (GECI) are used as a non-invasive alternative to measure calcium levels; being genetically expressed, no dye loading step is required and the protein is constantly replenished which is favorable for long term live cell imaging. Another advantage of genetically encoded sensors is the potential for tissue or organelle targeting, which can be achieved by modifying the promoter region or tagging with a localization signal moieties. For example, the ER targeted GECI D1ER was used to measure ER calcium levels in primary hippocampal astrocytes (Williams et al., 2013).

**Figure 7:**
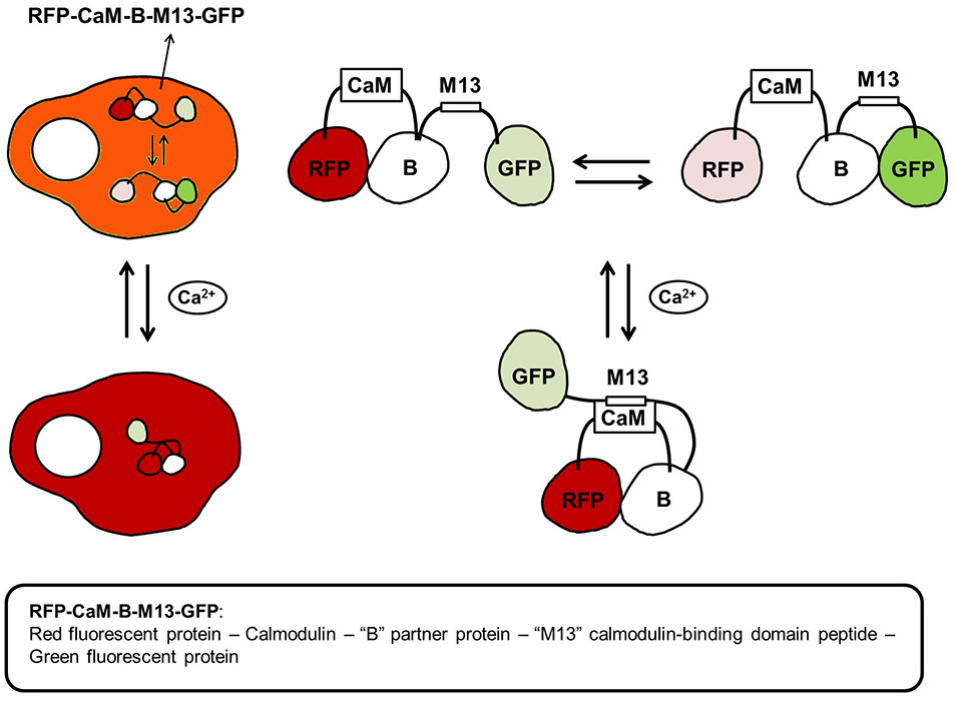
**A tripartite calcium ion biosensor**. The tripartite calcium biosensor relies on the expression of one plasmid-encoded polypeptide: a red, dimerization-dependent fluorescent protein (RFP) linked with calmodulin (CaM), a non-fluorescent partner protein “B,” the calmodulin-binding domain of skeletal muscle myosin light chain kinase (M13), and a green, dimerization-dependent fluorescent protein (GFP). In the absence of intracellular calcium (Ca^2+^) increase, “B” binds with either RFP or GFP, and the ratio of red-to-green signal is in equilibrium. Upon an intracellular increase in calcium (Ca^2+^), CaM binds Ca^2+^ and undergoes a conformational change, allowing it to bind M13. The binding of CaM with M13 causes a shift in the binding equilibrium of “B,” and an increase in the red-to-green ratio of the biosensor. Thus, cells expressing the calcium biosensor display red and green fluorescence in equilibrium in the absence of intracellular calcium release. Following an increase in calcium levels, red fluorescence increases in the cytoplasm.

GECI follow two design paradigms: (1) Single fluorescent protein based or (2) FRET-based sensor. Single fluorescent protein based sensors report calcium concentrations based on the intensity of a single fluorophore. The most popular single fluorescent protein based calcium sensors belong to the GCaMP family (Nagai et al., 2001). Recently, a family of ultrasensitive GCaMP calcium sensors have been developed that outperformed other sensors in cultured neurons and in zebrafish, flies and mice *in vivo* (Chen et al., 2013). GCaMP6 sensors can detect synaptic calcium transients in individual dendritic spines and have proven to be extremely useful for investigations on the organization and dynamics of neural circuits over multiple spatial and temporal scales. FRET-based sensors detect calcium concentration based on ratiometric measurements. An interesting ratiometric tripartite FRET-based Ca^2+^ sensor is shown below (Figure 7).

A recently optimized family of FRET-based ratiometric GECI “Twitch” showed greater signal amplitude and signal-to-background ratio compared to commercially available synthetic calcium dye Fura-PE3. The overall FRET of Twitch sensors upon calcium binding was improved from 10–20% ratio change initially to >1000%. As with other GECIs, a trade-off can be seen in Twitch sensors between high-affinity binding and fast response kinetics; sensors with lower affinity such as Twitch-5 (K_d_ = 9.25 μM, τ = 0.16 s) show relatively fast kinetics, whereas the higher affinity binding Twitch-3 (K_d_ = 250 nM, τ = 1.5 s) shows slower kinetics. Twitch-2B was able to measure tonic action potentials in mouse visual cortex neurons *in vivo*, exhibited low cytotoxicity and high signal to noise ratio after 141 days post transfection, demonstrating its usefulness as an *in vivo* calcium sensor (Thestrup et al., 2014). Since the quantification of calcium depends on ratiometric measurement between a donor and acceptor molecule, FRET-based GECI are less sensitive to artifacts arising from variability in sensor expression, laser intensity, cell thickness, and are preferred for long-term *in vivo* studies. Detailed procedures describing the calibration, common pitfalls, and organelle specific quantification of calcium levels using FRET-based ratiometric GECIs' was recently published (Park and Palmer, 2015a,b,c).

Advantages of single fluorescent protein GECI are a defined narrow optical spectrum and smaller construct size. A photoactivatable GCaMP GECI was recently designed for selective activation of GECI inside individual cells (Berlin et al., 2015) providing a powerful tool for investigations of neuronal signaling and synaptic plasticity. Recently, an elegant approach was proposed for monitoring brain activity with protein voltage and calcium sensors (Storace et al., 2015). Results from their studies suggest that the voltage sensor ArcLight has certain advantages over GCaMP6 calcium sensors such as optical electrophysiology of mammalian neuronal population activity *in vivo*.

### Bioengineered sensors for the detection and measurements of pH

In neural cells, as with most other cell types, pH is tightly regulated since both structure and function of cellular proteins depends critically on changes in pH, and changes in pH drive post translational protein modifications, such as phosphorylation. Phosphorylation of protein substrates changes their physical properties (e.g., charge), function (activation and inactivation) and intracellular fate (e.g., translocation from the cytosol to the nucleus and vice versa). Furthermore, detecting changes in H^+^ is critical for the function of sensory neurons which use acid-sensing ion channels for nociception and mechanoreception (Omerbašic et al., 2015; Sluka and Gregory, 2015). For instance, extracellular acidification to a pH of 6.9 generates a rapid inward current from acid-sensing ion channels (Waldmann et al., 1997). Properties of acid-sensing ion channels are reviewed elsewhere (Gründer and Pusch, 2015; Krishtal, 2015).

To monitor changes in pH, researchers have turned to fluorescent dyes and fluorescent proteins (probes and sensors). For some studies, it is important to correlate changes in pH with changes in some other intracellular process, such as adenosine triphosphate (ATP) levels. For these studies, one can use dual probes with non-overlapping excitation and emission wavelengths (Tantama et al., 2011). For example, in diabetes research, it has been shown that one can use a GFP-ATP sensor in conjunction with a RFP- pH sensor to measure ATP and cytoplasmic pH simultaneously in glucose deprived cells. Dual biosensors that exploit fluorescent dyes are superior to single pH measurements, but they are more difficult to generate (Fisher and Campbell, 2014). Among the most common measurements of pH changes relate to the endosomal and lysosomal vesicles. The difference in pH between an early endosome and a lysosomes is enormous and can cover 2 pH units corresponding to approximately a 100-fold difference in proton (H^+^) concentration (Paroutis et al., 2004).

### Bioengineered sensors for the detection of lactate

Neurons and other cells with high energy demand (e.g., heart) become functionally impaired when metabolic processes that normally produce ATP are disrupted. For example, the buildup of lactic acid can lead to neurodegeneration (Ruffin et al., 2014) but can also be neuroprotective when acting on GPR 81 or possibly related receptors (Lauritzen et al., 2014; Tang et al., 2014; Morland et al., 2015; Mosienko et al., 2015). L-lactate is produced by both neurons and astrocytes; moreover, there is a strong evidence that neurons use L-lactate as a supplementary fuel and signaling molecule, and genetically encoded fluorescence nanosensors exist to monitor energy metabolites, such as lactate (Sotelo-Hitschfeld et al., 2015). These sensors are valuable new tools to investigate the lactate pools in models such as dissociated astrocytes in cultures, cortical slices and even *in vivo*. The result obtained with these sensors show that astrocytes *in vitro* and *in vivo* maintain a cytosolic reservoir of lactate, which in response to plasma membrane depolarization, is immediately released into the extracellular space through a lactate-permeable ion channel. These findings support the roles for lactate in neuronal fueling and in gliotransmission (Sotelo-Hitschfeld et al., 2015).

### AuNP-based biosensors for detection of glucose

A good example of a simple AuNP-based colorimetric assay is that used to detect biological thiols (Ghasemi et al., 2015). The low-molecular-weight biological thiols show high affinity to the surface of AuNPs; this causes the replacement of AuNPs' shells with thiol containing target molecules, leading to the aggregation of the AuNPs through intermolecular electrostatic interaction or hydrogen-bonding. As a result of the predetermined aggregation, AuNPs' color and UV-visible spectra change. The principle of AuNP-based aggregation assays (with an example of protease detection) is illustrated in Figure 3. These AuNP-based colorimetric assays have been used to detect oxidative stress in neurons and non-neuronal cells (Kumar et al., 2013; Wang et al., 2015b). Glucose not only represents the primary energy source for the brain, but also plays important roles in synaptic transmission. In this assay, the aggregation of AuNPs was induced by glucose through cascade reactions involving glucose, H_2_O_2_, and ^*^OH (Jiang et al., 2010).

## Neuronal and glial responses to nanostructures

### Neural stimulation using nanostructures

Recent developments in NP technologies provide new approaches for recording and stimulating nerve cells. Among these are the incorporation of carbon nanotubes (CNTs) to improve implantable three-dimensional (3D) microelectrode arrays (MEA) to record nerve activity in large numbers of neurons in regional circuits (Gabay et al., 2007; Kim et al., 2014; Monaco and Giugliano, 2014). The advantage of using CNTs in the design of MEAs is that CNTs are chemically inert and stable. Furthermore, CNTs exhibit excellent electrical conductivity and, most importantly, biocompatibility with neurons (Lin et al., 2009; Gerwig et al., 2012; Musa et al., 2012; David-Pur et al., 2014; Samba et al., 2014). When embedded in polymer film, these CNT electrode arrays provide a flexible device to record activity that can be implanted in the brain.

In addition, promising new developments with NPs have demonstrated the feasibility of using heat generated from targeted NPs to control the activity of specific populations of neurons within a particular brain region. In one approach, neurons were stimulated with localized heat from gold nanorods (AuNRs) after irradiation with NIR laser light (Paviolo et al., 2014; Nakatsuji et al., 2015). Briefly, AuNRs are plasmonic nanoparticles that absorb minimally invasive NIR light and achieve highly localized photothermal heat generation (Paviolo et al., 2014). AuNRs can be targeted to the plasma membrane by coating them with a genetically cationized form of high-density lipoprotein (HDL; Nakatsuji et al., 2015). Not only does this complex reduce the cytotoxicity of the AuNRs but it also localizes the generated heat to neurons to enable the activation of heat-sensitive membrane ion channels, such as transient receptor potential vanilloid (TRPV) family members (Tominaga et al., 1998). This interesting approach has been used to stimulate cultured mouse sensory neurons from dorsal root ganglia, neurons that express TRPV1 endogenously, yet, this method has not been applied to neurons *in vivo*. One limitation is the poor penetration of NIR in intact neural tissue.

An interesting alternative technique uses magnetic nanoparticles (MNPs) to generate heat. Briefly, MNPs convert alternating magnetic fields into biological stimuli by dissipating heat through hysteretic power loss. Low-radiofrequency alternating magnetic fields (100 kHz to 1 MHz) can penetrate into the body without substantial attenuation and thus enable signal delivery into deep brain regions. Moreover, radiofrequency (RF) magnetic fields can be applied remotely, allowing for non-invasive remote stimulation of neurons in awake behaving animals (Huang et al., 2010; Chen et al., 2015). In addition to neuroscience, targeted magnetic NPs are being investigated in cancer therapy; as well, this method can be applied to manipulate remotely signal transduction pathways and other cellular machinery (Bonnemay et al., 2015).

The feasibility of using magnetic hyperthermia to stimulate neurons was demonstrated using coated manganese ferrite (MnFe_2_O_4_) MNPs conjugated with streptavidin (Huang et al., 2010). These particles were targeted to the surface of cultured hippocampal neurons overexpressing TRPV1. When exposed to low RF magnetic fields, the MNPs generated sufficient heat to activate the TRP channels and depolarize neurons without causing cell damage (Huang et al., 2010). This work established magnothermal stimulation as an attractive non-invasive method to excite specific neurons.

There has been some concern, however, that MNPs conjugated with proteins could become internalized and/or reduce the effectiveness of targeting and heat dissipation *in vivo*. To overcome these potential issues, Fe_3_O_4_ MNPs replaced MnFe_2_O_4_ MNPs. Untargeted Fe_3_O_4_ MNPs that have been optimized for efficient heat dissipation at clinically relevant alternating magnetic fields (Chen et al., 2015). These Fe_3_O_4_ MNPs have high heating rates, and when exposed to therapeutically relevant frequencies, they can trigger widespread firing of cultured hippocampal neurons expressing TRPV1. An attractive aspect of magnetothermal stimulation is its ability to stimulate neurons in deep brain structures; a good example is use in stimulating neurons in the ventral tegmental area (VTA; Chen et al., 2015). Since VTA neurons do not express TRPV1 channels endogenously, neurons were infected with lentivirus expressing TRPV1 cDNAs, the region was injected with Fe_3_O_4_ MNPs, and the animals were exposed to alternating magnetic fields. Even after 1 month of MNP injection, magnetic field stimulation triggered a significant increase in neural activity in the vicinity of the MNP injection site, as indicated by immediate early gene c-fos expression (Chen et al., 2015). This work demonstrates the feasibility of remote, wireless magnetothermal stimulation to activate neurons in deep brain areas.

Both NIR-AuNR and RF-activation of MNPs provide interesting approaches for stimulating neurons. However, this method can be applied only to neurons that express heat-sensitive ion channels, either endogenously, such as peripheral sensory neurons, or after expression with virally-mediated gene transfer. To enhance these approaches, it might be attractive to adapt them for use in exciting new implantable wireless fluidic devices that have been developed for programmable *in vivo* pharmacology (Jeong et al., 2015), thereby providing an interesting alternative to established optogenetics techniques (Warden et al., 2014). Optogenetic approaches and their attractions and limitations have been extensively reviewed elsewhere (Williams and Deisseroth, 2013; Thompson et al., 2014; Fan and Li, 2015; Kale et al., 2015; Lüscher et al., 2015; Tonegawa et al., 2015; Webber et al., 2015).

### Glial response to nanostructures

Collectively, glial cells (microglia and astrocytes) are equipped with sophisticated sensing, transducing and amplifying machinery that outperforms any artificial sensors. Microglia and astrocytes use toll–like receptors (TLRs) to sense pathogen signals, and those from nanoparticles. TLRs will recognize the “stranger” (e.g., nanoparticle) similar to the recognition of a pathogen (e.g., bacteria; Hanke and Kielian, 2011; Okun et al., 2011; Harry, 2013; Schaefer, 2014). TLR4 recognizes lipopolysaccharide (LPS) produced by Gram-negative bacteria and also nanoparticles associated with LPS (Lalancette-Hébert et al., 2010). TLR4 responds transiently to cerium oxide NPs (nanoceria). In contrast, unprotected (“naked”) CdTe QDs cause a strong microglia activation leading to robust luciferase activation as shown *in vivo* in transgenic mice expressing luciferase driven under the control of glial fibrillary acidic protein promoter (Maysinger et al., 2007). Similarly, when microglia are exposed to AuNPs, the intensity and temporal pattern of the TLR2 responses varies with the configuration of the NP. For example, in transgenic mice, gold nanorods exert a bimodal activation of microglia in transgenic mice with TLR2 promoter-luciferase reporter (Hutter et al., 2010).

Once TLRs have “sensed” the nanoparticle and other danger signals, the transduction system becomes engaged. This system includes IkB kinases, MAP-kinases and a number of transcription factors such as NFkB, AP-1 and interferon regulator factor (IRF) families (Takeuchi and Akira, 2010). Following the initial detection and transduction stages, signals recruit highly inducible genes, such as cytokines (e.g., interleukine 1beta, tumor necrosis factor alpha and others), which serve as amplifiers of the inflammation program (Glass et al., 2010). Under pathological conditions, microglia commonly assume a macrophage phenotype; however, in the healthy central nervous system, microglia do not behave as macrophages and their precise functions are currently under intense investigation (da Fonseca et al., 2014; Chen and Trapp, 2015; Tremblay et al., 2015).

## Challenges and limitations

### Fluorescent dyes

Fluorescent molecules (“dyes”) have played a major role in intracellular sensing and imaging in neural and other cells, in large part, because they respond rapidly to stimuli, have high signal intensities, and enter neural cells non-invasively. Consequently, these molecules are considered the current standard method for the quantification of intracellular analytes (Haugland, 2005; Lakowicz, 2006); none the less, some limitations compromise their usefulness. These include: (1) Possible interference with cellular processes and cytotoxicity; (2) organic solvents required for the dissolution of lipophilic probes; (3) unpredictable cellular responses when dyes interact with intracellular constituents; (4) relatively rapid bleaching limits their usefulness for time lapse experiments; (5) Only few dyes allow for ratiometric measurements.

### QDs

Although the photophysical advantages of QDs for experiments at the single cell level or *in vivo* experiments are greater than those of fluorescent dyes, the current generation of QD-based sensors has some drawbacks. For example: (1) the highest quantum yields are from QDs that contain toxic components, such as Hg; (2) QDs that emit signals in the near infrared spectrum are currently too large to enter neural cells unless their surfaces are adequately modified QD-based sensors, (3) and most QDs are not readily eliminated from the body and accumulate in liver and kidneys.

Ideas to reduce or prevent QD toxicity have been reviewed recently (Winnik and Maysinger, 2013). The extent of QD toxicity depends on its core composition, size, shape, surface coating, ligand arrangement, and charge (Hoshino et al., 2004; Jiang and Asryan, 2008; Verma and Stellacci, 2010; Kauffer et al., 2014). The mechanisms that account for the toxicity have not been fully resolved. They may involve the formation of reactive oxygen species (ROS) due to the degradation of the QD core and the release of free cadmium ions (Derfus et al., 2004), followed by oxidative stress and inflammation (Lovric et al., 2005; Manke et al., 2013). Or, exposure to QDs may cause cell growth inhibition and lipid peroxidation (Choi et al., 2007) as well as epigenetic and genetic changes (Choi et al., 2008; Stoccoro et al., 2013).

### AuNP and other NPs

AuNP-based sensors and enzymatic assays are not widely used and exist mainly as basic research tools. Colorimetric assays have a good potential for high-throughput applications: they are robust, simple, inexpensive, and require minimal instrumentation. However, colorimetric measurements are not always easily adapted for complex biological environments, such as tissues or living organisms, due to the substantial interference from cellular macromolecules. The detection limits of AuNP-based assays are surprisingly low, suggesting remarkable sensitivity and are promising for high throughput screening of enzyme inhibitors and activators. For measurements of enzymatic activities employing AuNPs in cells, FRET- based assays are more suitable then colorimetric measurements (Freeman et al., 2013; Lindenburg and Merkx, 2014; Chou and Dennis, 2015; LaCroix et al., 2015; Shamirian et al., 2015).

The ratio of acceptor to donor fluorescence is usually used as a surrogate for actual FRET efficiency. FRET efficiency can also be inferred from the rate of photobleaching of the donor or acceptor. Both of these approaches are qualitative and difficult to quantitate because the concentrations of fluorophore at the specific intracellular site is unknown. A more sophisticated approach, one that overcomes the problem of inconsistent fluorophore concentrations is fluorescence life time imaging (FLIM), an approach that relies on lifetimes of fluorophores and QDs (Murakoshi et al., 2008; Ueda et al., 2013; Doré et al., 2014; Chen et al., 2014b; Datta et al., 2015; Kaur et al., 2015; Yellen and Mongeon, 2015).

With any nanoparticle-based sensor, of course, it is important to take into consideration the possibility of nonspecific binding and ligand exchange. For example, in the case of AuNPs, with thiolated ligands, the ligands can be exchanged with intracellular glutathione. To prevent ligand exchange and nonspecific binding, AuNPs can be PEGylated. The main challenge still is monitoring the enzymatic processes *in vivo*, in a longitudinal, real-time manner; for these purposes, cell type specific expression of genetically engineered proteins remains a promising alternative.

## Looking ahead to new ways to sense neural cells under physiological and pathological conditions

Many pharmacological interventions in neurological disorders fail because the initiation of intervention started too late and the damage to the tissues was too extensive and irreversible. One of the reasons for such a failure is the lack of adequate tools to detect the changes in neural and other cells early enough when pathology can be arrested and deleterious consequences minimized. Nanosensors are beginning to change the diagnostic arena but they are still at an early stage of development. Nanoparticle-based and bioengineered sensors as well as fluorescent molecular probes should be used in a complementary manner. New nanoparticle-based lipase sensor sparked interest in development of sensors for other lipases and could be used to reveal the role of lipids in neurodegeneration (Tang et al., 2015).

As opposed to the well-studied and most frequently used spherical particles, many other particle morphologies have optical resonances in the near-infrared spectral window, allowing a deeper penetration within tissues and an absence of photochemical damage; these features are highly advantageous for intracellular or *in vivo* imaging applications. Only a few designs employing gold nanorods (Cheng et al., 2015; Park et al., 2015; Zhang et al., 2015a) or nanocages (Chen et al., 2005a,b) exploit the tunability of surface plasmon absorption peak and optimize the sensitivity of AuNP-based assay.

For two-photon luminescence (TPL) gold nanorods and other anisotropic morphologies should be exploited. TPL imaging, which is superior to dark-field imaging in terms of signal-to-noise ratio, is an attractive option for intracellular imaging, where a strong background signal often hampers the detection of low concentrations of AuNPs. TPL has been employed mainly for bioimaging (Vo-Dinh et al., 2013; Yellen and Mongeon, 2015), but it should be considered for detection of changes in enzymatic activities in neural cells under physiological and pathological conditions. Luminescent AuNPs with high quantum yields are attractive candidates to replace QDs or some organic fluorophores (Maysinger and Hutter, 2015). A “plasmonic resonance energy transfer (PRET)” can be exploited for measurements of enzymes engaged in disrupted redox homeostasis, neuroinflammation and protein shedding (cleavage of the ectodomain of membrane proteins; Altmeppen et al., 2013; Saftig and Bovolenta, 2015).

Nanoparticle-based and bioengineered sensors presented here were mostly tested in non-neural and some neural cells but their employability under true pathological conditions remain to be investigated. Currently available sensors require considerable improvements to provide reliable, reproducible and simple measurements of biomarker concentrations, duration of the processes and their precise location. The internalization of nanoparticle-based sensors still remains a challenge.

Nanosensing is a very dynamic field, and the employability of the new designs and the proportions of targeted application areas keep changing. In the near future, advances in nanotechnology and imaging techniques combined with electrophysiological recordings, could elucidate critical signaling players under physiological and pathological conditions thereby providing the way of more successful testing of new therapeutic interventions in neurological disorders. Combined approaches employing electrophysiology, bioengineering and nanotechnology could contribute to finding ways of getting “out of clutter and finding simplicity” (Albert Einstein).

### Conflict of interest statement

The authors declare that the research was conducted in the absence of any commercial or financial relationships that could be construed as a potential conflict of interest. The reviewer Ruxandra Vidu and handling Editor declared a current collaboration and the handling Editor states that the process nevertheless met the standards of a fair and objective review.
